# Life cycle assessment (LCA): informing the development of a sustainable circular bioeconomy?

**DOI:** 10.1098/rsta.2020.0352

**Published:** 2021-09-20

**Authors:** Eva Sevigné-Itoiz, Onesmus Mwabonje, Calliope Panoutsou, Jeremy Woods

**Affiliations:** Centre for Environmental Policy (CEP), Imperial College London, (ICL), 18-19 Princess Garden, South Kensington, London SW7 1NE, UK

**Keywords:** LCA, life cycle assessment, biobased systems, stakeholder engagement, circular bioeconomy, biomass value chain

## Abstract

The role of life cycle assessment (LCA) in informing the development of a sustainable and circular bioeconomy is discussed. We analyse the critical challenges remaining in using LCA and propose improvements needed to resolve future development challenges. Biobased systems are often complex combinations of technologies and practices that are geographically dispersed over long distances and with heterogeneous and uncertain sets of indicators and impacts. Recent studies have provided methodological suggestions on how LCA can be improved for evaluating the sustainability of biobased systems with a new focus on emerging systems, helping to identify environmental and social opportunities prior to large R&D investments. However, accessing economies of scale and improved conversion efficiencies while maintaining compatibility across broad ranges of sustainability indicators and public acceptability remain key challenges for the bioeconomy. LCA can inform, but not by itself resolve this complex dimension of sustainability. Future policy interventions that aim to promote the bioeconomy and support strategic value chains will benefit from the systematic use of LCA. However, the LCA community needs to develop the mechanisms and tools needed to generate agreement and coordinate the standards and incentives that will underpin a successful biobased transition. Systematic stakeholder engagement and the use of multidisciplinary analysis in combination with LCA are essential components of emergent LCA methods.

This article is part of the theme issue ‘Bio-derived and bioinspired sustainable advanced materials for emerging technologies (part 1)’.

## 1. Applying LCA to guide the development of a sustainable circular bioeconomy

During the last 50 years, life cycle assessment (LCA) has evolved to include well-established and internationally accepted standards such as ISO 14040-14044 [1,2], impact models and applications in multiple sectors (e.g. energy, agriculture, etc). Application of LCA and life cycle-based approaches has been extensively used to support policy formation, starting from defining policy agendas, assisting in defining policy targets and providing evidence for monitoring and impact assessment on products and services [1]. Companies and industries have used LCA in product and process development; marketing, development and selection of indicators to monitor the environmental performance of products or plants; selection of suppliers or subcontractors; and strategic planning. Finally, LCA has also been applied for guiding and informing consumers through ecolabels and public reporting from industry and academia [3–5].

The rapid expansion in the application of LCA relies on its ability to operate across value chains and its data-driven operation. It quantifies the inventory flows, input and outputs based on measurements of mass and energy balance, e.g. the relationship between emissions or resource consumption and impacts based on proven causalities or on empirically observed interactions [4]. Applying a life cycle perspective across a broad range of environmental and socio-economic issues allows comparative assessments of products and processes, identification of trade-offs and prevention of burden shifting between life cycle stages, processes and impact categories that are taking place in different locations at different times [4]. Moreover, LCA allows for sensitivity analyses when technological pathways of low technology readiness level are involved and can facilitate the identification of hotspots where future optimization and research efforts must focus [6]. Such characteristics make LCA approaches suitable to inform decision-making during the transition to a sustainable circular bioeconomy which focuses on the sustainable, linear and circular resource-efficient valorization of biomass in integrated, multi-output production chains (e.g. biorefineries) while also making use of residues and wastes and optimizing the value of biomass over time via cascading [7].

However, environmental LCA also present some inherent limitations related to complex modelling, blurred or inconsistent boundary setting, short-time horizons during which the outputs are considered to be valid, budget, data availability, trends in politics, research, lack of well-established impact models and selective or partial application of LCA methodology. Any or all of these limitations can be a reason for controversy and rejection of LCA results [8,9]. These are particularly important for the assessment of biobased systems that are often complex combinations of technologies and practices over geographically dispersed and long distances and with wide and uncertain sets of indicators [10]. The indicators may also be sensitive to land use patterns and change over time, to biomass supply logistics, to the combinations of highly innovative and conventional technologies deployed in the value chain [11], to the production and use of by- and co-products and to cultural preferences (e.g. diet or amenity value of landscapes) as discussed below.

Social life cycle assessment (S-LCA) approaches can improve inclusiveness and broaden the scope of bioeconomy [12,13] to include the social dimension, especially linkages to food, materials consumption, energy access and mobility [14]. Most social analysis methods so far apply a mix of participatory approaches and qualitative analysis at local level with national to global level-specific quantification of limited performance metrics such as child labour and corruption [15,16]. While bottom-up analysis with the contribution of local stakeholders facilitates prioritization of needs and the assessment of impacts at the level of implementation, it risks narrowing the scope and excluding impacts which could occur in neighbouring or other regions around the world. Combining local stakeholder methods with S-LCA holds promise for obtaining a more complete view of the social issues related to developing bioeconomies [17,18].

S-LCA is recognized as the social equivalent to environmental LCA [19]. It can operate from cradle-to-grave and addresses social impacts within specified stages of a value chain at local and global scales [20]. Its systemic approach aligns well with biomass supply and value chains for bioeconomy integration at the territorial level or in specific sector-product systems [14,17,21,22]. It has limitations however: (i) in S-LCA the cause and effect chains are difficult to correlate, with regard to production activities and their potential social effects, making it challenging to select appropriate indicators [23], (ii) the development of quantitative databases for S-LCA lags those of environmental LCA and the analysis relies on local data collection or publicly available statistics on criteria which have been determined to be generally important (e.g. gender equality, child labour, forced labour, clean energy access) [23], (iii) lack of harmonized protocols for collection of data on value chain-specific indicators.

Life cycle costing (LCC) has also been suggested as a consistent framework for combining LCAs and economic assessments [24,25]. The methods follow the usual unit-cost approach across value chain stages but also assess the effects on societal welfare caused by exchanges that would otherwise not be accounted for (externalities) [26]. In biobased value chains, this facilitates grouping of budgets costs, transfers and externalities, in each biomass supply and value chain stage and identification of respective physical and economic parameters.

This paper aims to evaluate the role of LCA in addressing challenges linked to informing the transition toward a sustainable circular bioeconomy, discusses how certain methodological limitations can be improved to better inform decision-making and synthetizes improvements needed to resolve future development challenges. All three sustainability pillars, environment, society and economy as well as the policy relevance of LCA approaches to resolve systemic challenges in biobased value chains are considered.

## 2. Land use and biomass production

### (a) Competition for land and biomass

Land is a finite and increasingly contested resource. Land use changes driven by increased expectations of future demands for the biomass needed to supply the bioeconomy and a growing, more affluent population, have raised concerns over competition and displacement of current activities [27,28]. LCA includes the impacts arising from the competition for land and consequential indirect land use change (ILUC) which differs from direct changes in the use of the land (i.e. direct land use change (DLUC)). Impacts associated with DLUC include carbon and nutrients loss from soils, soil erosion, water consumption and loss of biodiversity [29]. ILUC occurs when land used for e.g. food and feed production changes to biomass production for materials and/or energy, [28–31] potentially driving DLUC in other areas [29].

ILUC, however, cannot be directly observed and measured in a given place and time. To overcome this, studies have applied various approaches to model and simulate ILUC [31]. Still, the majority of LCA on bioenergy and biomaterials does not include ILUC mostly due to the modelling complexity, diversity of drivers behind ILUC, the uncertainty related to their assessment and the disagreement among experts about how to allocate the resulting impacts [29,32]. The scientific community agrees that LUC needs to be evaluated within LCA approaches, but the lack of a consistent framework remains a challenge.

Feedstock diversification through the valorization of waste and residual streams, exploitation of low quality land, yield improvement, improved conversion efficiencies, carbon cascading, etc. are considered to be important potential options for limiting competition for land and biomass [32]. However, new feedstock, strategies and technologies may imply burden shifting from one stage to another, from one impact category to another, and also competition for biomass that can lead to further indirect economic and social consequences. Engaging with biomass producers through S-LCA and stakeholder validation approaches can steer focus to locally sourced biomass, associated skills and infrastructures. The process will provide valuable insights on how to build on local capacities, design and implement improved sustainable supply (e.g. through agroecology, crop rotation, etc.) and resource-efficient value chains (e.g. broaden market opportunities for residues that remain on field after harvesting the main product, etc.) within safe planetary boundaries while delivering economic return and social resilience to rural regions. In due course, S-LCA may support analysis to identify regions where local comparative advantage lies in biomass production and the associated trade-offs with long-distance transport, land use change and conflict, value retention and social development.

### (b) Spatial differentiation and ecosystem services in life cycle assessment

A major challenge in LCA is spatial differentiation in life cycle impact assessment (LCIA) methods. This is because land is spatially dependant and heterogenous and impacts resulting from land use are site-dependent and closely related to natural resource availability (i.e. water and land) and ecosystems quality [33]. Harmonization of LCIA regionalization [34,35] and land use characterization modelling has advanced considerably over the last two decades with approaches including water scarcity and degradation [36–41], soil quality [42–44] or biodiversity [45–47].

Ecosystem services (ES) have also been integrated in LCA studies because of their importance for the sustainable circular bioeconomy not only in terms of ecological sustainability and the intrinsic value of biodiversity, but also in ensuring high productivity, maintaining regenerative capacity and ensuring the resilience of biobased production systems [9,48–52].

However, no LCIA method is truly complete today. There are gaps in impact categories including plastic pollution or invasive species and in the coverage of potential environmental impacts in LCIA. There is a need to increase the number of impact pathways considered by developing new methods or improving existing methods [53]. In addition, regionalization is partially limited by a lack of data formats, poor site-dependent inventory data availability e.g. soil quality indicators, and a lack of software that supports regionalization [54], which are critical steps to informing about land use impacts and full application of LCA in decision-making.

### (c) Accounting for the temporal dimension

**Figure 1.**
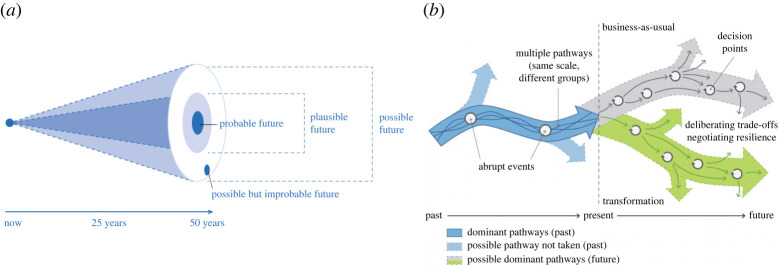
The cone of possible futures and different types of futures and pathways (left hand side fig: [55]; right hand side fig: [56]).

Environmental impacts arising from the growth and use of the biomass and its products are also temporally heterogenous with temporal discontinuities associated with evolving practices and technological innovations arising alongside stochastic events that can affect entire biomass value chains ([55]; figure 1). The treatment of time in LCA (i.e. the inclusion of temporal dynamics in LCA) is another area where guidance is urgently needed. Recent approaches allow the inclusion of time considerations in LCA, but this remains uncommon and is mostly handled on a case-by-case basis [57,58]. The special feature of biomass-based systems is the uptake of short cycle atmospheric carbon and its accumulation in the feedstock, which can be accounted as negative flow in the GHG-emissions profile, and then, this negative flow is neutralized in case of a CO_2_ emission in the end of life treatment (e.g. as a result of combustion and re-release to the atmosphere) [57–59]. However, time separation between carbon uptake and release in end-of-life treatments can be long and the accounting of biogenic carbon storage depends on the product's durability but also on the land management practices used in generating the feedstock [32].

Recent studies have challenged the carbon neutrality hypothesis by introducing metric indicators to assess the global warming potential of biogenic carbon [57,60] also because biobased systems introduce a number of additional time-dependent components such as field maturation and yield changes, changes in soil organic matter and soil carbon stocks, technology and process changes over the length of the time horizon which can be decades or centuries, market response times and carbon payback period [4].

The need to capture time-sensitive indicators such as biogenic carbon flows along the product lifetime is especially relevant for the GHG performance of biomass value chains, and hence, the temporal scope of the study is critical [32]. In addition, time consideration is also relevant in other life cycle stages and life cycle phases including LCI and LCIA [58]. Although traditionally LCA studies have disregarded the accounting of biogenic carbon and time in the evaluation of bioenergy and biomaterials [29,57,58,61–63], the inclusion of temporal dynamics in LCA is needed to reduce uncertainties and increase representativeness. It is also advisable to perform full LCA including end of life, as well as conduct scenario analysis by considering alternative waste treatment options [32] as a way to overcome these challenges.

## 3. Biomass processing and its conversion to biobased products

### (a) Emerging technologies: a forward look

While the fossil-based industry is based on mature, optimized technologies, the viability of many biobased products is still dependent on emerging and novel technologies [4,63]. These novel bioproducts inherently involve complex interactions between variable raw materials, processes, conversion technologies, products and services. Their commercialization is still nascent and most of them are not produced on a large scale [61] including pigments, battery electrodes or composites. Progress in biotechnology, technological learning, scaling-up and process integration of biobased refineries into existing petrochemical ones are expected to reduce production costs, improve environmental performance and increase social awareness about the benefits of biobased products [27,61,64]. LCA can guide the possible co-location of biorefineries with existing petrochemical refineries [64] by analysing scenarios of preserving and using existing petrochemical infrastructures, process and technologies in relation to new biobased supply chains and emerging technologies. LCA can also be applied to promote sustainability at early stages in the development of a technology or suite of technologies in a value chain [58], influence design and ensure that sustainability goals of innovation are achieved [65] and not compromised. Possible limitations to this include lack of data, incumbents against which to compare, and uncertainty with respect to both how the emerging technology will be deployed as well as the market conditions into which the technology will be deployed [65].

To overcome some of the difficulties associated with LCAs at early laboratory stages, different approaches including the modelling of extreme scenarios, learning curves, predictive scenarios, scenarios range, Bass modelling, product cannibalization analysis, etc have been presented in recent literature [65,66]. However, there is still a lack of systematic guidance on how to address the particular challenges of emerging technologies within the context of emerging markets [65]. In addition, low-bulk and high-value applications such as biochemicals and biopharmaceuticals or products derived from GMO or engineered plants are underrepresented in the LCA literature and more studies are needed to fill this gap [32].

The most difficult challenge is the projection of the future that involves considering subjective judgements and ideas based on the present moment against anticipated perceptions of what a sustainable future might encompass. The future can be a projection of historical trends (i.e. business as usual) or what could happen (i.e. preferable, likely or unlikely); it can be a plausible and probable future based on current trends and knowledge about how the world works and how innovation in technologies and knowledge could evolve; it could also be an unanticipated future. Projections of the future need to encompass the potential impacts of stochastic and abrupt events such as the COVID-pandemic and even the amount of woody material that can arise from natural disturbance [67], that are inherently difficult to anticipate and therefore the decisions and effects that derive from them give rise to unexpected or even those currently perceived to be implausible futures (figure 1).

Recent studies have presented methodological suggestions on how LCA can be applied for evaluating the sustainability of emerging systems, helping to identify environmental and social opportunities prior to large R&D investments. Accessing economies of scale and improved conversion efficiencies while maintaining compatibility across a broad range of sustainability indicators and public acceptability remains a key challenge for the bioeconomy. LCA can inform, but not by itself resolve this complex dimension of sustainability. Suggested solutions include, among others, the use of a broad multidisciplinary systems analysis including techno-economic assessment (TEA), market assessment, systems modelling, future studies, behavioural characterization and expert elicitation [65].

## 4. Stakeholder engagement in life cycle assessment

Falcone [68] states that social assessment, compared to environmental and economic assessments, may involve a broader spectrum of aspects that directly affect stakeholders, ranging from human rights, working conditions, health and safety issues, equity, social responsibility, job creation and social participation in defining and generating social capital in present and future generations, to access to basic resources, and happiness [69]. Therefore, stakeholder perspectives must be incorporated when formulating the key criteria for inclusion in social sustainability assessments for bio-based products [70]. Stakeholder participation in the sustainable, circular bioeconomy is a topic increasingly addressed in the academic literature [71]. The overarching aim of stakeholder involvement is to better understand their perceptions, opinions and likely impacts on their personal growth and quality of life [72]. Mattilla [18] suggest that identifying local stakeholder values and comparing them with the data availability from databases could highlight relevant subcategories for further studies. S-LCA is often combined with multicriteria decision support analysis (MCDA) to better define biobased value chains and the indicators that interpret their performance [73].

Stakeholder engagement is also relevant in environmental LCA as it can contribute to ensure that key questions and concerns are addressed, identify barriers and unintended consequences of the system under study and to provide a platform for discussion and mutual learning [8]. Stakeholders can also be important sources of spatially and temporally resolved data and practices. However, in the development of biobased products, and in general in environmental LCA studies, efforts to engage stakeholders are not yet standard practice. There are only small samples of studies and projects (e.g. BESTER project [74]) that have attempted to integrate LCA with stakeholders' participation [74–78]. It is evident that modelling decisions in LCA including system boundary definition, functional unit and impact category selection, and weight determination all have consequences for the outcome of individual LCA results. When exploring scenario uncertainty jointly with the identification of financial, technological and social barriers carefully targeted stakeholders will need to engage in the co-design and production of the LCA [32].

## 5. Relevance of life cycle assessment to policy

The transition to biobased economy is the cornerstone of political aspirations in several strategies, including the USDA's Biopreferred programme [79], the European Green Deal [80] and other initiatives [81–83] which acknowledge the importance of shifting from fossil-based goods and services to biobased, climate-compatible consumption patterns and behavioural choices [56]. Biobased value chains require systemic analysis to understand how interventions, coordinated across the value chain stages, can overcome challenges and resolve gaps. The use of LCA can therefore support progressive change throughout the whole system by focusing the policy agenda towards more sustainable options and quantifying their performance in comparison to the prevailing market counterfactuals, reference product and service systems. Synergies with other initiatives of relevance should be further considered and leveraged, including interfaces between chemicals, products and waste legislation [83].

**Figure 2.**
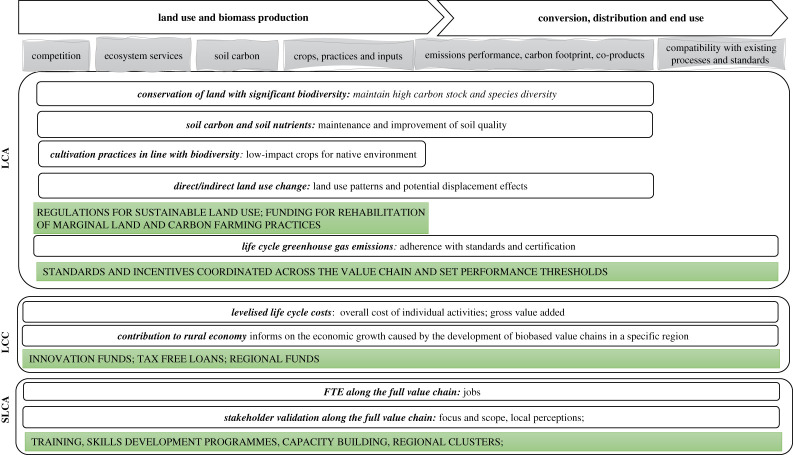
Impact categories and relevant policy interventions (in green) that can be informed by LCA approaches across biobased value chains.

Figure 2 outlines important challenges across the biobased value chain stages and groups the relevant impact categories based on the LCA method used for their assessment (LCA, LCC and S-LCA). It identifies potential policy interventions that can be integrated in a coordinated manner across the value chain stages to improve their performance and facilitates resolving the challenges.

Future policy interventions that aim to promote the role of the bioeconomy and focus support on strategic value chains that can efficiently exploit local raw materials and infrastructures can benefit from the systematic use of LCA. In this way, LCA can provide important insights on the impact categories across biomass supply and value chains and impact hotspots, alongside the provision of informed evidence about the range of potential values through policy-relevant indicators [84]. Moreover, LCA can improve the accuracy of impact results by accounting for spatial variability in production technologies and environmental factors. The nuanced findings can be valuable to policy makers for prioritizing support to resource efficient, locally sourced value chains. The analysis can also be used to optimize potential co-benefits and minimize trade-offs when mobilizing domestic biomass including the mitigation of biomass competition, and facilitation of rural, industrial, and economic development, and the simultaneous delivery of higher biomass shares within sector targets [85].

## 6. Conclusion

### (a) Resolving future development challenges

LCA can be used in multiple roles to inform the transition to a sustainable and circular bioeconomy, as shown in table 1. However, as highlighted in this paper, critical challenges remain in using LCA to inform private and public policy. These challenges include LUC quantification, time and spatial considerations including effects on soil, ES and biogenic carbon accounting, new approaches to evaluate emerging technologies and the integration of economic and social assessment within the SLCA framework. Other studies also indicate that transparency, scenario analysis, sensitivity and uncertainty analyses along with early stakeholder engagement can enhance the robustness of LCA results [8]. Similarly, the integration of multiple environmental impacts is needed to avoid burden shifting and different LCA modelling principles can facilitate decision-making in different contexts and scales (e.g. consequential or attributional LCA) [8,9,27–29,32,61].

**Table 1.** Biobased value chain stages challenges, the role of LCA and steps to address LCA limitations.

bioeconomy value chain stages	transition challenges	role of LCA	steps to address LCA limitations and challenges
land use	— competition for land — ES — soil carbon	— quantification of environmental/ economic/social impacts and trade-offs — quantification of soil impacts	— agreement & methodology development (e.g. LUC, carbon storage, time, ES, regionalization LCIA, opportunity cost of land, etc) — methodological consensus (e.g. allocation) — more data, data connectivity and software support for regionalization — integration with other tools/approaches — stakeholder engagement; capacity building; increased awareness
biomass production/logistics	— competition for biomass — suitable selection of species and sustainable crop practices	— quantification of environmental/ economic/social impacts and trade-offs — hot spots identification and plant location	— agreement and methodology (e.g. time, ES, regionalization LCIA, profitability, etc) — methodological consensus (e.g. allocation) — integration with other models/approaches — stakeholder engagement
conversion	— technological readiness level — gaps between R&D and deployment of technologies	— promote sustainability at early stages — influence design — identification of environmental opportunities prior to large R&D investments	— integration with tools (e.g. techno-economic assessment (TEA), market assessment, systems modelling, future studies, behavioural characterization, MCDA, etc.) — methodological improvements for time consideration and upscaling — more LCA of emerging technologies and products (e.g. biopharmaceuticals or products derived from GMO) — stakeholder engagement
distribution/end use	— compatibility of biobased products and services with existing processes, standards and distribution channels — lack of consumer awareness about quality (sustainability) and availability of biobased products	— development and selection of indicators used in the monitoring of environmental performance — LCA dissemination and benchmarking	— standardization and harmonization — scenario, sensitivity and uncertainty analysis — stakeholder engagement

However, there remains a real risk that rushing into defining new impact categories, more exposure compartments, more complexity, more of everything, may result in undermining the value and credibility of LCA [8]. The systematic iteration with stakeholders from the outset of an LCA is necessary to ensure societal needs are properly addressed. In addition, the integration of LCA with appropriate tools to create synergies that can influence robust and positive decision-making across various markets, supply and values chains are proposed as more efficient approaches to ensuring the credibility and robustness of the findings.

Lessons learnt by LCA practitioners working on biobased systems can help improve LCA modelling principles for informing the transition to a biobased economy. However, this will imply additional effort to support consensus building, new methodological improvements alongside cross-sectoral and methodological harmonization. In this regard, important harmonization efforts have been carried out with the development of Product Environmental Footprint (PEF), other European Standards such as the EN 16760 [86] and the initiation of a Global Life Cycle Impact Assessment (GLAM) by Life Cycle Initiative. These initiatives have been developed to facilitate quantification and comparability of environmental impacts across multiple products and technological systems as well as to promote coherence and consistency in the performance of LCAs. Nevertheless, they still do not provide explicit guidance on most of the aspects highlighted in this paper and although some of these limitations can be prevented if methodological choices and best practice are commonly followed in accordance with ISO standards and ILCD guidelines [87], further agreement and more case studies are needed to evaluate its wide applicability. For example, the promotion of waste streams and biorefineries is at the heart of the bioeconomy, but for LCA to support decision-making between the full range of technological and feedstock supply options, consensus and consistent practices on how to account for coproducts and circularity in LCA is urgently needed.

Other initiatives on harmonization on data collection, quality and data sharing such as GLAD [88] or Bonsai [89] are in progress and needed efforts that the LCA community is taking to develop the mechanisms and tools to ensure change throughout the whole sector to guide a material and successful biobased transition. More of these initiatives are needed but both consensus building and harmonization are challenging because of the multiple options, views on fundamental choices and their alignment or not, with researchers' and societal preferences. Wide and open discussions in the literature and other arenas (e.g. online LCA discussion fora) illustrate that the LCA community is aware of the lack of harmonization and the need for flexibility in the face of very broad heterogeneity but opposite views continue to exist. This represents a major future challenge in LCA, although elucidating the steps to overcome this situation is out of the scope of this paper, the authors aim to provide LCA practitioners with guidance on how to improve robustness and wide applicability of LCA. The authors also aim to provide decision-makers with a framework to interpret LCA results in the context of policy design and implementation so as to improve the environmental information available for consumers.

## Data accessibility

This article has no additional data.
